# Heart Rate Variability Characteristics in Sedentary Postmenopausal Women Following Six Months of Exercise Training: The DREW Study

**DOI:** 10.1371/journal.pone.0002288

**Published:** 2008-06-04

**Authors:** Conrad P. Earnest, Carl J. Lavie, Steven N. Blair, Timothy S. Church

**Affiliations:** 1 Pennington Biomedical Research Center, Louisiana State University System, Baton Rouge, Louisiana, United States of America; 2 Department of Cardiology, Ochsner Health System, New Orleans, Louisiana, United States of America; 3 Department of Exercise Science, Arnold School of Public Health, University of South Carolina, Columbia, South Carolina, United States of America; 4 The University of North Texas, Denton, Texas, United States of America; University of Las Palmas de Gran Canaria, Spain

## Abstract

**Background:**

Decreased heart rate variability (HRV) is associated with a higher risk of mortality. Overall, postmenopausal women have lower levels of HRV than premenopausal women, which may be additionally complicated by lifestyle related behaviors such as physical inactivity and obesity. Though cardiorespiratory exercise training increases HRV, little is known regarding the exercise dose necessary to promote this improvement.

**Methodology/Principal Findings:**

Our primary aim was to measure HRV in post-menopausal women following 6-months of exercise training. We examined supine resting HRV in 373 post-menopausal women (45–75 y) after 6-months of randomly assigned and double-blinded administered exercise training exercise training at 50%, 100% and 150% of the NIH Consensus Development Panel's recommended minimal physical activity level. This corresponded to 4, 8, or 12 kcal/kg per week (KKW) of energy expenditure. At baseline, we observed no significant differences in HRV or hormone replacement use between treatment groups. However, we did observe that Caucasian women and those taking antidepressant medications had lower levels of baseline HRV. After 6-months of exercise intervention, we observed a dose dependent increase in all parasympathetically derived time and frequency domain measurements across exercise groups after adjustment for age, ethnicity, antidepressants, and baseline rMSSD (all, P<0.001). For example, the parasympathetic index rMSSD was greater than control (23.19±1.0) for the 4-KKW (25.98±0.8; P = 0.14), 8-KKW (27.66±1.0; P<0.05), and 12-KKW (27.40±0.0; P<0.05) groups at follow-up.

**Conclusions/Significance:**

Moderate intensity exercise training exercise is sufficient to improve HRV in previously sedentary postmenopausal women in a dose-dependent manner, as 4-KKW is insufficient to improve parasympathetic indices of HRV, while 12-KKW conferred no greater improvement than 8-KKW.

**Trial Registration:**

Clinicaltrials.gov NCT 00011193

## Introduction

Menopause represents a key transition period in a woman's health. A fundamental etiology associated with menopause is the intricate link between estrogen metabolism and the autonomic nervous system. Autonomic nervous system balance can be assessed non-invasively via the use of heart rate variability (HRV). While a thorough review of HRV can be obtained elsewhere,[1] HRV is clinically useful because reduced HRV is associated with an increased risk of CV disease, type II diabetes and insulin resistance and has been shown to be reduced in postmenopausal women as compared with premenopausal women [2], [3], [4], [5]. Equally, the assessment of HRV in postmenopause is important because cardiovascular (CV) disease typically occurs later in women than in men; yet, the fact remains that CV disease is one of the two leading causes of death in women [6], [7]. A lifestyle behavior that has a significant affect on the metabolic processes surrounding CV disease is cardiorespiratory exercise training.

Current recommendations from the National Institutes of Health (NIH) Consensus Development Panel recommend that women attain a minimal level of moderate intensity activity such as walking briskly for 30 minutes [8]. In a recent report, we observed significant improvements in peak aerobic power for varying doses of exercise in postmenopausal women obtaining ∼50%, 100%, and 150% of the NIH recommendation [9]. Cross-sectional reports, as well as reports in cardiac rehabilitation,[10] suggest that regular exercise training improves HRV; however, exercise training studies elaborating on the dose necessary to affect beneficial HRV changes are not clear [11], [12], [13], [14]. Limited exercise training studies conducted on postmenopausal women using small sample sizes (N<10) report no change in HRV after exercise training [12], [13], [14]. However, an observational study by Reland et al. has shown that postmenopausal women exhibiting high levels of physical activity also exhibit high levels of parasympathetic indices assessed by HRV [15]. Recently, we reported an improvement in HRV characteristics accompanying an 8-week exercise training intervention period [16]. Given the aforementioned observations, we hypothesized that HRV would exhibit a dose effect following 6-months of exercise training in postmenopausal women. To test our hypothesis, we examined the HRV response of women participating in the Dose-Response to Exercise in Women study (DREW) who participated in 6-months of strictly monitored, moderate intensity exercise training at approximately 50%, 100%, and 150% of the minimal NIH Panel's recommendation for physical activity.

## Methods

We have recently published a complete description of the DREW design, methods, and primary outcomes for our current cohort. These two papers detail how we derived the sample size for our main outcomes, the randomization sequence and allocation procedures, blinding techniques, recruitment and adverse events [9], [17]. In brief, DREW is a randomized, dose-response, double-blind, exercise training trial that complies with Declaration of Helsinki and comprised of a non-exercise control group and three exercise training groups exercising at incremental doses (50%, 100%, and 150%) of the minimal NIH Panel's recommendation for energy expenditure. The study was originally reviewed annually by The Cooper Institute and subsequently approved by the Pennington Biomedical Research Center IRB for the continued analysis and publication of pertinent research findings. Prior to participation, all participants signed a written informed consent document outlining the procedures involved in the DREW study. The primary outcomes for DREW study included peak aerobic power and resting blood pressure [9], [17]. The data presented herein is the result of a secondary analysis examining the potential HRV response to exercise training. The supporting CONSORT checklist for this trial is available as Checklist S1, and the protocol is outlined in [17].

After an initial run-in period, we randomized 464 postmenopausal women (45–75 y) to 1 of 3 exercise training groups or a non-exercise control for a 6-month intervention period. Study participants were sedentary (exercising<than 20 minutes; <3 d/wk; <8000 steps/d assessed over the course of 1 week), overweight or obese (BMI 25.0 to 43.0 kg/m^2^), and had a systolic blood pressure of 120.0 to 159.9 mm Hg. As previously reported, there was no difference in this cohort at baseline or following our intervention for fasting measures of cholesterol, triglycerides, and glucose [9]. We excluded women who had a history of stroke, heart attack, or any serious medical condition that prevented participants from adhering to the protocol or exercising safely. Following baseline testing, inclusive of HRV (see below), participants were randomized into respective treatment groups.

### Fitness Testing

We conducted our fitness testing using a Lode Excalibur Sport cycle ergometer (Groningen, Netherlands), an electronic, rate-independent ergometer. Participants cycled at 30 Watts (W) for 2 min, 50 W for 4 min, followed by increases of 20 W every 2 min until they could no longer maintain a pedal cadence of 50 rpm. Breath-by-breath respiratory gases were measured using a Parvomedics True Max 2400 Metabolic Measurement Cart. Volume and gas calibrations were conducted before each test. Gas-exchange variables (VO2, CO2 production, ventilation, and respiratory exchange ratio [RER]) were averaged every 15 s. Heart rate was measured directly from the ECG monitoring system. Ratings of perceived exertion (RPE) were obtained using the 20-point Borg scale. Fitness was defined as the mean of two exercise test assessments at both baseline and 6 months. The reproducibility of the two fitness tests (VO2abs) was examined and characterized by an intraclass correlation of 0.88 at both baseline and follow-up testing.

### Daily Physical Activity and Exercise Training

To assess potential changes in non-supervised physical activity, all randomized participants wore a step counter (Accusplit Eagle, Japan) to record their daily steps. Exercisers removed their step counter during supervised exercise sessions. Cardiovascular exercise training consisted of having women expend 4, 8, or 12 kcal/kg per week (KKW). During their exercise training sessions, women alternated exercise training using a recumbent cycle ergometer or treadmill. The target training intensity for each exercise training session was 50% of each woman's VO_2max_ for each exercise modality. We used a computer-controlled exercise training management that allowed us to input of relevant data points on each woman (week of exercise, KKW dose according to group assignment, their HR associated with 50% VO_2max_, training HR zone, body weight, and number of visits per week). The computer then provided us with the appropriate power output (PO) for the cycle ergometer, and the correct speed and grade for the treadmill. By knowing the exact PO for the cycle ergometer and the treadmill, the total kilocalories expended each minute and the time needed to reach the target energy expenditure for the exercise session or for the week can be calculated. The duration of each individual session depends on the number of visits required to reach the target KKW. To monitor the participant's caloric expenditure over the course of 6 months, we created a weekly tracking report, which was used to track and make calculate caloric adjustments. The number of calories expended per session was adjusted each week, within the limits of the study design so that the total number of calories expended is equal to the total number prescribed per week for the 24-wk program. This report also averaged the number of visits per week so that we can determine whether the participants are exercising at least two, but no more than four, sessions each week.

During exercise, HR is monitored every 3–6 min using a Polar HR monitor (Polar Vantage XL and Polar Vantage NV). The target HR was used to monitor each woman's performance during each session to ensure that she was exercising at the proper intensity. Monitoring HR allowed us to control exercise intensity and document the specific amount of exercise done during each session. As women improved their fitness, they worked at gradually higher PO and spent less time to expend the required KKW. We contacted participants if they missed a scheduled session so that arrangements could be made to bring them back on schedule as soon as possible. As previously reported, the non-exercise training control group maintained their current level of activity during the trial period and showed no significant increase in physical activity [9].Those women in the exercise training groups adhered well to their prescribed exercise training and attended 92% of their exercise training sessions [9].

### Heart Rate Variability

We examined HRV between 06:30 a.m. and 11:00 a.m. for 25 minutes in a semi-dark room (22–23°C) following a 12-hour fast. Participants abstained from consuming caffeine-containing products and alcoholic beverages for 12 hours and heavy exercise for 48 hours. We controlled respiration rate using a metronome at a fixed rate of 12 breaths/min (0.2 Hz). HRV measurements were conducted at the same time (±1 hour) of day for each assessment period. We used a two-channel ECG signal detected by a Polar Heart Rate Monitor and transmitted online to a PC through Polar Advantage Interface receiver. QRS timing was fixed at 1 ms. The computer program labeled each QRS complex, and the resulting signal was filtered to eliminate ectopic beats and artifact edited and replaced with an average value. Segments containing >15% artifact ectopy were interpreted as premature beats and were excluded from our data analysis [18]. We quantified HRV from the last 5 minutes of R–R interval recording.

For our primary outcome measurement, we examined the parasympathetic nervous system by calculating the square root of the mean of the sum of the squares of differences between adjacent R–R intervals (rMSSD). rMSSD is considered to be a stable measure of parasympathetic modulations in heart rate [1]. As a secondary measurement of mixed signaling, we also calculated the standard deviation of all R–R intervals (SDNN), which reflects the cyclic components responsible for variability in the period of recording and reflective of both sympathetic and parasympathetic tone. As a tertiary aim, we analyzed frequency domain in three log normalized frequency bands define as: high frequency power (HF; 0.15–0.40 Hz), low frequency power (LF; 0.04–0.15 Hz), very low frequency power (VLF; 0.0033–0.04 Hz), and total frequency power (PT; 0.00–0.40 Hz). The filtering techniques are described in previous reports [18], [19].

### Statistical analysis

We used a generalized linear model (GLM) to analyze the influences of the differing doses of exercise training on HRV characteristics. We adjusted all our outcomes among the randomization groups for select specified covariates including baseline HRV, race, and the use of antidepressant medication. When our GLM demonstrated a significant overall statistical effect and a significant treatment group effect, we further explored pairwise comparisons between the exercise training groups vs. the control group using a Dunnett-Hsu post-hoc assessment. The Dunnett-Hsu test allows for specific multiple pair-wise comparisons while still protecting against type I statistical errors. Between group differences at baseline and follow-up in prevalence were examined using Chi-square tests. We also used a subgroup analysis to compare dose-response effects across predefined baseline groups with significance of interactions relationships between variables were tested using a Spearman correlation analysis and denoted as r_s_. All reported P values are two-sided (*P*<0.05). All analyses were performed using SAS version 9.1 (Cary, NC).

## Results

### Baseline Heart Rate, Medication, and Physiologic vs. HRV Correlation Characteristics

**Figure 1:**
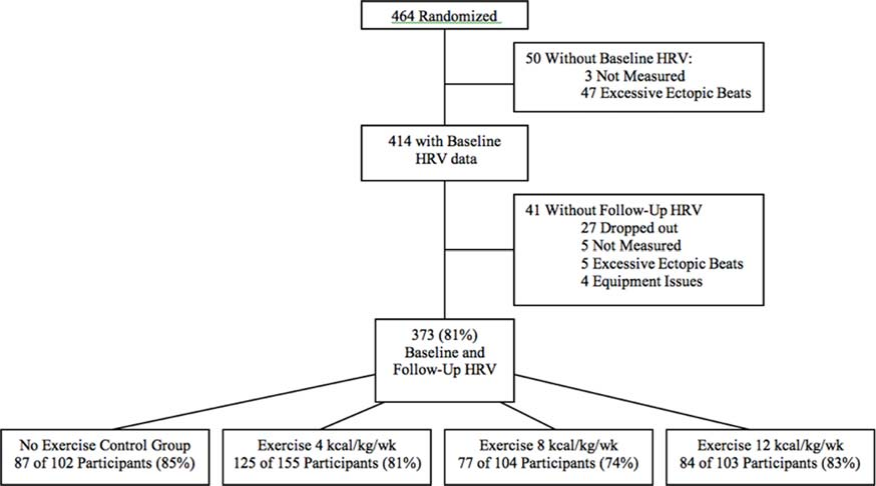
DREW Heart Rate Variability Assessment: Schematic diagram represents CONSORT flow chart of the DREW heart rate variability study analysis.

We have presented a schematic CONSORT flowchart our HRV analysis in Figure 1. Baseline health characteristics are presented in Table 1 and an examination of parasympathetic levels (rMSSD) by ethnicity, smoking status and medication use in Table 2. Overall, we were able to successfully obtain HRV measurements on 373 individuals at follow up. In our analysis of baseline characteristics (Table 2), we observed significant differences for ethnicity (*P*<0.0001) and antidepressant medication use (*P*<0.002). Post-hoc assessments revealed that African American women had statistically higher levels of rMSSD vs. Caucasian women (*P*<0.0001). Spearman correlations characteristics for various physiologic variables and HRV indices are presented in Table 3. With the exception of VLF power, we observed small yet significant correlations between age and all indices of HRV. When analyzing HRV change from baseline to follow-up we did not observe any significant correlations between most HRV parameters and changes in fitness (VO2 L/min), weight, and BMI. There were three exceptions as we did observe a negative correlation between BMI and SDNN (r_s_ = −0.1; *P*<0.05), VLF (r_s_ = −0.12; *P*<0.0004), and body weight (r_s_ = −0.11; *P*<0.02).

**Table 1:** Baseline Characteristics.

	Total Cohort (N = 373)	Control (n = 87)	4 KKW (n = 125)	8 KKW (n = 77)	12 KKW (n = 84)
Demographics					
Age, mean (SD) y	57.5 (6.3)	57.5 (5.8)	58.2 (6.4)	57.4 (6.4)	56.7 (6.3)
Ethnicity (%)					
Caucasian	67	68	65	62	72
African American	27	24	29	30	26
Hispanic or Other	6	8	6	8	2
Anthropometry					
Weight (kg)	84.5 (12.0)	86.4 (12.4)	83.3 (11.4)	85.2 (6.4)	83.8 (11.6)
BMI (kg/m^2^)	31.8 (3.7)	32.3 (3.9)	31.4 (3.6)	32.1 (3.9)	31.4 (3.6)
Waist circumference (cm)	101.2 (11.5)	103.4 (11.8)	100.2 (10.8)	102.5 (11.9)	99.2 (11.7)
Cardiovascular					
VO_2max_ (L/min)	1.31 (0.2)	1.34 (0.3)	1.29 (0.2)	1.29 (0.2)	1.33 (0.2)
Blood pressure (mmHg)					
Systolic	139.6 (13.0)	142.1 (12.3)	138.4 (13.2)	139.8 (13.7)	138.6 (12.9)
Diastolic	80.9 (8.6)	80.7 (7.9)	80.4 (9.1)	80.9 (8.6)	81.8 (8.5)
Hematology*					
LDL-C (mg/dL)	118.4 (26.4)	117.3 (25.9)	116.9 (27.6)	118.0 (25.4)	121.9 (26.3)
HDL-C (mg/dL)	57.5 (14.3)	56.9 (14.2)	58.1 (14.4)	57.3 (15.8)	57.2 (13.0)
Medication history (%)					
Antihypertensive	22	23	26	34	31
Thyroid	22	16	10	18	20
Antidepressant	19	18	18	18	21
Hyperlipidemic	22	16	18	14	11
Hormone Replacement Therapy	46	49	45	45	46
Smoking (%)					
Never	59	55	62	61	54
Past	36	39	33	35	38
Current	6	6	5	4	8

Abbreviations: LDL-C, low density lipoprotein cholesterol; HDL-C, high density lipoprotein cholesterol; HRT, hormone replacement therapy.

* To convert LDL-C and HDL-C to mmol/L multiply by 0.0259.

**Table 2:** Baseline Heart Rate Variability (rMSSD) Characteristics by Ethnicity, Smoking Habitus, and Medication Use.

	n	Mean (SD)	P
Ethnicity			<0.0001*
Caucasian	248	21.14 (10.2)	
African–American	102	27.13 (13.4)	<0.0001†
Hispanic or Other	23	24.09 (13.9)	
Smoking			0.47
Never	218	23.37 (12.1)	
Past	134	22.03 (11.0)	
Current	21	24.52 (11.3)	
Antihypertensive			0.26
Not taking	268	23.32 (11.9)	
Taking	104	21.87 (11.1)	
Thyroid			0.47
Not taking	314	23.09 (11.4)	
Taking	58	21.94 (12.9)	
Antidepressant			<0.002*
Not taking	302	23.85 (11.5)	
Taking	71	19.14 (11.6)	
Hyperlipidemic			0.6
Not taking	315	23.04 (11.5)	
Taking	57	22.21 (12.8)	
Hormone Replacement Therapy			0.16
Not taking	196	23.54 (11.3)	
Taking	170	22.03 (12.1)	

* Significant overall statistical effect.

† Significantly different than Caucasian.

Abbreviation: rMSSD; Root mean square successive difference of RR intervals.

**Table 3:** Baseline Relationship between Physiologic Characteristics and Indices of Heart Rate Variability.

	Indices of Heart Rate Variability					
Physiologic Characteristic	Time Domain		Frequency Domain			
Baseline	rMSSD	SDNN	HF	LF	VLF	Total
Age, y	−0.19^*^	−0.15^†^	−0.22^‡^	−0.11^§^	−0.07	−0.18^//^
VO_2max_, L/min	0.04	0.07	0.05	0.08	0.0003	0.06
Weight, kg	−0.02	−0.04	−0.02	−0.02	−0.04	−0.03
BMI, kg/m^2^	0.02	0.00	0.02	0.02	−0.04	0.01

Data represent Spearman correlation coefficients.

Statistical notations are: (^*^) *P*<0.0002, (^†^) *P*<0.003, (^‡^) *P*<0.001, (^§^) *P*<0.03), (//) *P*<0.0004.

Abbreviations: SDNN, standard deviation of RR intervals; rMSSD, Root mean square successive difference of RR intervals; HF, log high frequency spectral power (0.15–0.40 Hz); LF, log low frequency spectral power (0.04–0.15 Hz); VLF, log very low frequency spectral power (0.0033–0.04 Hz); PT, log total spectral power frequency.

### Resting Heart Rate and HRV Analyses

We present the HR, time, and frequency domain analyses in Table 4. All values represent the adjusted least squared means covaried for baseline value, age, antidepressant use, and ethnicity. Our primary GLM analyses for resting HR showed a statistically significant time effect for resting HR (*P*<0.0001), but not for treatment group effect (*P* = 0.10) after 6 months of exercise training. Thus, we found no specific exercise training effect despite a trend for a reduction in resting HR. For our time domain HRV measurements, we observed a significant main statistical effect for rMSSD and SDNN (*all*, *P*<0.0001) and for treatment group (*all*, *P*<0.006). Post hoc comparisons revealed that rMSSD for the 8-KKW and 12-KKW exercise training conditions were greater than the control after 6 months (*P*<0.05). However, our post-hoc assessment only showed a significant statistical difference in SDNN for the 8-KKW treatment versus control (*P*<0.05).

**Table 4:** Heart Rate Variability Outcome Measures at Follow-Up.

	Control (n = 87)	4 KKW (n = 125)	8 KKW (n = 77)	12 KKW (n = 84)
Heart Rate (b/min)	64.8 (5.0)	63.9 (4.0)	63.0 (4.0)	63.2 (6.0)
Time Domain (ms)				
rMSSD^*, †^	23.19 (9.3)	25.98 (8.9)	27.66 (8.8)^‡^	27.40 (9.2)^‡^
SDNN^*, †^	32.93 (10.1)	34.96 (10.1)	37.84 (8.8)^‡^	36.20 (10.1)^‡^
Frequency Domain (ms^2^)				
HF_Ln_^*, †^	5.20 (0.9)	5.40 (1.1)	5.56 (1.0)^‡^	5.49 (0.9)^‡^
LF_Ln_^*, †^	5.21 (0.9)	5.46 (1.1)^‡^	5.63 (1.0)^‡^	5.53 (0.9)^‡^
VLF^*, †^	4.64 (0.9)	4.94 (1.1)^‡^	5.03 (1.0)^‡^	4.88 (0.9)^‡^
LF∶HF Ratio	1.60 (2.0)	1.55 (2.2)	1.64 (2.0)	1.41 (1.8)
Total_Ln_^*, †^	6.28 (0.9)	6.49 (1.1)^‡^	6.61 (1.0)^‡^	6.55 (0.9)^‡^

Data are mean (SD).

Analyses adjusted for age, ethnicity, and antidepressant medication use.

Statistical notations: (^*^) significant overall main effect (*P*<0.0001); (^†^) significant group effect (*P*<0.006); (^‡^) significantly different from control (*P*<0.05).

Abbreviations: SDNN, standard deviation of RR intervals; rMSSD, Root mean square successive difference of RR intervals; HF, log high frequency spectral power (0.15–0.40 Hz); LF, log low frequency spectral power (0.04–0.15 Hz); VLF, log very low frequency spectral power (0.0033–0.04 Hz); PT, log total spectral power frequency.

For the frequency domain analysis, we observed a significant main statistical effect for the HF_Ln_, LF_Ln_, VLF, and PT components (*all*, *P*<0.001) and for treatment group (*all*, *P*<0.002). Post hoc assessment revealed that the HF index of HRV denoting parasympathetic tone was significantly greater versus the control group for the 8-KKW and 12-kcal KKW treatment conditions (*both*, *P*<0.05). Post hoc comparisons for LF, VLF, and PT showed each HRV index to be greater than the control group for the 4-KKW, 8-KKW, and 12-KKW treatment conditions (*all*, *P*<0.05). No statistical effects were noted for the LF∶HF ratio.

## Discussion

The primary finding from our study is that moderate intensity exercise training improves HRV characteristics in previously sedentary, overweight or obese postmenopausal women. To our knowledge, this is the largest clinically controlled exercise training trial examining this question and the only study examining the dose response of exercise training and HRV. Of particular interest is that these effects appear to be dose-related regarding parasympathetic tone (i.e., rMSSD and HF) as only women exercising at or above the NIH recommendation exhibited a positive change in these HRV characteristics. The same pattern was present for SDNN; however, SDNN represents mixed sympathetic and parasympathetic signaling which makes the interpretation of this index more difficult. These findings have important implications for women entering into and completing menopause.

Normal aging influences a variety of indices related to CV circulation including the adrenoreceptor responsiveness and baroflex sensitivity [20], [21]. As it pertains to HRV, parasympathetic tone shifts to a lower range [22], [23]. Though parasympathetic tone is generally higher in women than men, aging reduces the difference between the two genders whereby changes in HRV begin approximately at menopause [7], [24], [25]. A hallmark feature of exercise training is an improvement in CV dynamics, inclusive of an improvement in the neuroregulatory aspects of cardiac regulation through the sinoatrial node [26], [27], [28], [29]. Further, high levels of cardiorespiratory fitness are associated with higher HRV characteristics in endurance-exercise training young and older men as denoted by various indices including LF, HF total power, SDNN, and rMSSD [30], [31], [32], [33]. However, this relationship is not as clear in older women where some studies show no difference in HRV characteristics, while others report an improvement in women exhibiting higher physical activity levels [14], [15], [34]. This is important clinically as a moderate level of physical activity is recommended for an aging population during a time when physiologic function and physical activity levels are waning [35], [36]. Our findings affirm the theory that physical activity facilitates improvements HRV in older women and that the quantity of exercise training necessary to affect such an improvement is relatively modest.

Previous cross-sectional studies helped to define the question evaluated our the current study. In a recent report, Reland et al. categorized older women into three groups: low activity (1000 kcal/week), moderate activity (>1000–2000 kcal/week), and high activity (>2000 kcal/week) and found that women in the high activity group who participated in chronic physical activity demonstrated an increase in parasympathetic indices of HRV [15]. A recent prospective study by Britton et al. also found that women who avoided being placed in the lowest/adverse quartile of HRV function and HR during 5.6 years of follow-up are most strongly influenced by baseline exercise training, body mass index, cholesterol, and blood pressure [37]. While these investigations support the concept that physical activity improves HRV, they do not adequately reflect the efficacy of using exercise training as an intervention to improve HRV.

Earlier literature describing the use of exercise training to modulate HRV in women is limited and difficult to interpret due to variances in study protocol participant age, length, small sample sizes and the exercise training intensity used for the intervention. Davy et al reported that 12 weeks of aerobic exercise at 70% of maximal heart rate (∼50–55% VO_2max_) was insufficient to improve HRV in *eight* postmenopausal women [12]. In contrast, Ito et al observed a significant improvement in HRV characteristics following 8 weeks of exercise training in mildly obese younger women (45.9±4 y; [38]) In the only study we are aware of using a longer exercise training intervention, Stein et al observed an improvement in HRV characteristics for older women (66±4 y) exercising at 70% of VO2_max_ (∼80–85% HR_max_). From these studies, one could surmise that intensity and study length are important factors for examining HRV changes in women; however, the results of these trials may create confusion regarding the effectiveness of exercise training for improving HRV in women.

Perhaps the most simplistic explanation surrounding previous findings is that these trials were performed with small sample sizes and may have been statistically underpowered to adequately identify changes in HRV relative to exercise training. In our current trial, parasympathetic tone significant increased following 6-months of exercise training at 8-KKW and 12-KKW. The two most relevant points to be garnered from our findings are (1) that a significant change in HRV does accompany longer periods of exercise training consistent with the NIH recommendation and (2) a greater level of energy expenditure may not be necessary to achieve greater improvements in HRV. In essence, our findings suggest a “threshold effect” regarding aerobic exercise training and improvements in HRV. Nonetheless, we caution readers not to misinterpret that 4-KKW is ineffective for improving HRV as several indices of HRV were increased at the 4-KKW. Unfortunately, the interpretation of these results is more difficult to explain regarding the parasympathetic influence associated with HRV. SDNN and LF power are thought to represent mixed sympathetic and parasympathetic signaling and the physiologic characteristics surrounding VLF are still a matter of debate [1]. It has also been suggested that VLF is a “dubious” measurement when using short-term recordings ≤5 minutes [1]. With respect to the actual energy expenditure associated with exercising at 4-KKW, changes in HRV may take a longer period to fully realize. A final point to consider is whether an improvement in HRV is actually protective. Specifically, does an improvement in HRV carry with it the inherent assumption that the modification of HRV translates directly into CV protection [1]? The paradox of this question exists whereby it is largely accepted that exercise training decreases CV mortality and regular exercise training is capable of modifying autonomic balance [39], [40], [41], [42].

We believe that improvements in HRV via exercise training are likely to reduce the risk of mortality for several reasons. In animal studies, dogs documented with a high mortality risk due to the previous occurrence of ventricular fibrillation during acute myocardial ischemia were randomly assigned to 6 weeks of either daily exercise training or cage rest. After exercise training, indices of HRV increased by 74% and all animals were better able to survive an experimental ischemic challenge after the exercise training period [43]. In humans, exercise training also accelerates the recovery sympathovagal interaction and improves patient prognosis in post-MI patients [44], [45]. Though each of the aforementioned studies has been reported in animal and humans with CV disease, perhaps the most compelling argument for protection is the observation that CV disease mortality is associated with a constellation of risk factors [46], [47]. Thus, even if modulating HRV through exercise training does not directly affect mortality, per se, the practice of CV exercise influences multiple risk factors associated with CV disease due to its broad influence and on both the ANS and CV system.

A primary strength of the DREW study is that it used a well-controlled dosage of exercise, with a high level of adherence. Moreover, the exercise dose used in our study is easily obtainable in sedentary women. The DREW study has limitations because its sample is limited to sedentary postmenopausal women. We do not know if the results will apply to other women or men. An interesting finding from our study is that women exercising at a higher caloric expenditure (12-KKW) did not experience greater improvements in HRV than women exercising at 8-KKW. Finally, our findings should not dissuade women from exercise training at a lower dose of they are not able to achieve the minimum level of exercise training necessary to improve HRV. Given the global effects of aerobic exercise training, HRV represents only one measure of CV risk, which may ultimately show improvement accompanying longer periods of exposure.

## Supporting Information

Checklist S1 CONSORT Checklist
(0.23 MB PDF)
